# Heredity, Health, and Personal Beauty

**Published:** 1891-07

**Authors:** 


### HEREDITY, HEALTH, AND PERSONAL BEAUTY. By John V. Shoemaker, M. D., Philadelphia (1231 Filbert Street) and London. F. A. Davis, Publisher. 1890. Price, cloth, $2.50; half morocco, $3.50, net.

This work, which consists of a series of chapters devoted to the various physiological aspects of man in relation to the absorbing questions of heredity, aims to bring the factors of organic evolution to bear upon the subject.

This is unquestionably the proper spirit in which to set out on so difficult a path, and one that will more often light the darkness of this undiscovered country than cast a greater gloom.

All truly thinking men of to-day approach the great human problems from the standpoint of evolution and environment.

Dr. Shoemaker is, happily, a staunch advocate of the effect of surrounding conditions upon the organism in changing its characters and bringing it into harmony with its environment, and of the transmission of such characters to the offspring. All characters are, in a way, acquired ones, and, in time, become congenital through the agency of natural selection. It is a mere "war of words" to argue the question on the distinctions of such terms. The view of Weismann is untenable and insufficient to account for the facts, and this Dr. Shoemaker has clearly set forth in his introduction.

The work is undoubtedly in the right direction of thought, and will help to bring thinking people to the proper conception and view of human life. Health and beauty, as two of its greatest factors, must be brought to a thoroughly sound biological basis, and in this the author has done a good work.

